# Developmental exposure to triclosan and benzophenone-2 causes morphological alterations in zebrafish (*Danio rerio*) thyroid follicles and eyes

**DOI:** 10.1007/s11356-022-24531-2

**Published:** 2022-12-10

**Authors:** Maximilian Kraft, Lisa Gölz, Maximilian Rinderknecht, Johannes Koegst, Thomas Braunbeck, Lisa Baumann

**Affiliations:** Aquatic Toxicology and Ecology Section, Centre of Organismal Studies, University of Heidelberg, Im Neuenheimer Feld 504, 69120 Heidelberg, Germany

**Keywords:** UV filter, Disinfectant, Endocrine disruptor, OECD guidelines, Histopathology, Transgenic line

## Abstract

Thyroid hormones (THs) regulate a multitude of developmental and metabolic processes, which are responsible for vertebrate development, growth, and maintenance of homeostasis. THs also play a key role in neurogenesis of vertebrates and thus affect eye development, which is vital for foraging efficiency and for effective escape from predation. Currently, there are no validated test guidelines for the assessment of TH system-disrupting chemicals (THSDCs) in fish. Consequently, the present study was designed to demonstrate the suitability of novel thyroid-related endpoints in early life-stages of fish. Embryos of a transgenic zebrafish (*Danio rerio*) line expressing the reporter gene *tg:mCherry* in their thyrocytes were used to investigate the effects of the environmental THSDCs triclosan (TCS, antibacterial agent) and benzophenone-2 (BP-2, UV filter) on thyroid follicle and eye development. Both BP-2 and TCS caused thyroid follicle hyperplasia in transgenic zebrafish, thus confirming their role as THSDCs. The effect intensity on follicle size and fluorescence was comparable with a 1.7-fold increase for BP-2 and 1.6-fold for TCS. Alterations of the cellular structures of the retina indicate an impact of both substances on eye development, with a stronger impact of TCS. With respect to guideline development, results provide further evidence for the suitability of morphological changes in thyroid follicles and the eyes as novel endpoints for the sensitive assessment of THSD-related effects in fish.

**Supplementary Information:**

The online version contains supplementary material available at 10.1007/s11356-022-24531-2.

## Introduction 

Testing of endocrine-disrupting chemicals (EDCs) has received rapidly increasing attention in recent years. These substances can have a strong impact on the different endocrine axes of vertebrates such as the hypothalamic-pituitary–gonadal (HPG) or the hypothalamic-pituitary-thyroid (HPT) axis, which regulate essential physiologic, metabolic, and developmental processes (Demeneix and Slama 2019). Adverse effects on, e.g., the HPT axis range from (neuro) developmental disorders (Cediel-Ulloa et al. 2022) to cancer (Marotta et al. 2020). EDCs are under strict regulatory control and need to be tested for their possible impacts to protect both environment and human health (ECHA & EFSA 2018). Gaps in testing of potential EDCs were identified by the European Commission (EC) and OECD (Organisation for Economic Cooperation and Development; Holbech et al. 2020), and, therefore, new tests and approaches are currently being developed and optimised. A recent effort to promote this research was taken by the EC, which launched 8 projects under Horizon 2020 Research and Innovation Actions (Topic: new testing and screening methods to identify endocrine-disrupting chemicals). Among these projects, ERGO (“EndocRine Guideline Optimization”) aims to combine human health and environmental safety assessments of EDCs, which are currently considered separately (Holbech et al. 2020). As all other endocrine modalities are currently assessed in fish, ERGO aims to demonstrate that fish (e.g., zebrafish, *Danio rerio*) are fully adequate and effective models to assess thyroid hormone system disrupting (THSD) effects by identifying, evaluating, and integrating suitable new endpoints into already existing OECD test guidelines.

To establish new THSD-related endpoints in fish, a comprehensive knowledge of the HPT axis and its regulatory functions is indispensable. In vertebrates, thyroid hormones (THs) play a crucial role in regulating different essential processes, such as development (e.g., of the nervous system), cell growth and differentiation, and metabolism (Bernal 2007). In fish, THs are crucial for the metamorphosis from larvae to juveniles as well as related developmental processes (Power et al. 2001; Porterfield and Hendrich 1993) such as inflation of the swim bladder (Cavallin et al. 2017, Stinckens 2018 & 2020). Moreover, THs play a key role in eye development: they have an impact on the formation of the optical nerve, on the development of photoreceptors, retina, and on eye size (Bohnsack & Kahana 2013; Bertrand et al. 2007; Heijlen et al. 2014; Suzuki et al. 2013). Since THSDCs can interfere with different parts of the HPT axis, various changes in different structures of the eye may occur. If such changes affect visual performance, consequences can be fatal, since eyesight is essential for food detection, orientation, or predator avoidance (Besson et al. 2020). A recent AOP (adverse outcome pathway) developed by Gölz et al. (2022) describes the chain of events leading from molecular inhibition of the enzyme thyroid peroxidase (TPO) to altered visual function via altered retinal layer structure in fish (https://aopwiki.org/aops/363). Based on these considerations and the importance of the eye for survival, the disruption of eye size, retinal structures, and changes of vision-related gene transcripts can be considered as possible meaningful endpoints for assessing environmentally relevant adverse effects of TH system disrupting chemicals (THSDCs; Dang et al. 2021; Gölz et al. 2022).

The zebrafish embryo has proven to be a valuable model for research into THSD-related effects and especially eye malformation (Baumann et al. 2016 & 2019). Since zebrafish eye morphology and physiology are well comparable to other vertebrates including humans, extrapolation of effects makes zebrafish a promising model for the detection of THSDCs in other vertebrates (Gestri et al. 2012). The fast and rapid development of the eyes and the related visual system in zebrafish offers additional practical advantages for investigations of THSD-related effects: The retinal layers are already established as early as 50 h post-fertilisation (hpf; Schmitt and Dowling 1999), and the first thyroid follicles can be identified at 55 hpf (Alt et al. 2006). Thus, the analysis of eye and thyroid follicle development in THSDC-exposed zebrafish embryos can be performed within the standard time frame (120 h) of the fish embryo toxicity test (FET; OECD TG 236), i.e., testing is restricted to developmental stages not regarded protected according to current EU animal welfare legislation (EU 2010; Strähle et al. 2012).

Furthermore, THs are required for the migration of cells of the neural crest into the head region and are, therefore, important for eye morphogenesis in zebrafish. Accordingly, THs play a key role for craniofacial and ocular morphogenesis (Bohnsack & Kahana 2013; Cohen et al. 2022). Since thyroid hormone receptors (THRs) are known to be located in the outer nuclear layer of the fish retina (Bertrand et al. 2007) and photoreceptor cone development is at least partly regulated by THs (Suzuki et al. 2013), THSDC exposure can have adverse effects on eye development (Gölz et al. 2022). Morphological changes of the retinal layers in THSDC-exposed zebrafish were, e.g., observed by Reider and Connaughton (2014), who described significant changes in the diameter of different retinal layers after methimazole exposure. THSDCs were also found to inhibit the expression of the outer segment (OS)-specific genes, which lead to the failure of forming this important retinal layer (Xu et al. 2015). Baumann et al. (2016 and 2019) observed multiple pathological alterations of the eye, such as a reduction of eye size and pigmentation of the retina in THSDC-exposed zebrafish embryos, which could be related to transcriptional changes in the eyes and behavioural changes of exposed embryos.

Based on this background, the present study was designed to analyse morphological changes in thyroid follicles and histopathological alterations in selected layers of the retina in zebrafish embryos exposed to environmentally relevant THSDCs. This approach combines mechanistic and apical endpoints in a comparably short test system, and the use of non-protected embryonic stages is in line with the 3R principle (replace, reduce, refine; Russel & Burch 1959).

As a proof-of-concept, two environmentally relevant compounds were selected to demonstrate the suitability of the test system: the ultraviolet (UV) filter benzophenone-2 (2,2′,4,4′-tetrahydroxybenzophenone, BP-2) ranges among the most widely used UV filters, which are used in many personal care products, such as sunscreens, lipsticks, perfumes, hairsprays, and shampoos (Blüthgen et al. 2012). In rivers, levels of up to 300 µg/L have been measured (Ramos et al. 2015). BP-2 is highly photostable and lipophilic, making it a relatively persistent chemical that can affect aquatic organisms. In fish, benzophenones may cause DNA damage (Cuquerella et al. 2012) as well as endocrine disruption (Kunz et al. 2006). Given the scarcity of studies, the exact mode-of-action (MoA) for endocrine disruption is unknown. In 5 d old zebrafish embryos, Fong et al. (2016) detected malformations including a shortened tail, blood clotting, and enlargement of the yolk sac due to BP-2 exposure. In both a human thyroid follicular cell line and fish, BP-2 has been shown to inhibit thyroperoxidase (TPO) activity and to reduce 3,3′,5-triiodo-L-thyronine (T_3_), and tetra-iodinated thyronine/thyroxine (T_4_) levels (Song et al. 2012; Schmutzler et al. 2007).

The second THSDC used in this study, triclosan (2, 4, 4′-trichloro-2′-hydroxy-diphenyl ether; TCS), is a synthetic antimicrobial agent widely used in home and personal care products (Fu et al. 2019) as well as in veterinary and industrial applications. Since the worldwide use of antibacterial products is steadily increasing, TCS has not only been detected in surface water, sediments, and soil, but also in aquatic organisms and even human body fluids and tissues (Zhu et al. 2016). Especially due to the massive increase in the use of disinfectants during the COVID-19 pandemic, the amount of TCS released into the environment has received growing attention in ecotoxicological research. TCS is known to be toxic for aquatic organisms, because it impairs embryonic development and hatching, alters enzyme activities, causes genotoxicity and mortality in fish embryos as well as alterations in swimming behaviour and survival of adult fish (Pinto et al. 2013). Chronic low-dose exposure to TCS may cause sublethal effects in aquatic organisms such as endocrine disruption and developmental impacts (Wang and Tian 2015). As for BP-2, the MoA of TCS in humans and wildlife is still poorly investigated: In mammals, TCS reduces circulating TH levels and alters the activity and gene expression of enzymes related to TH homeostasis (Crofton et al., 2007). In rats, TCS led to histopathologic changes in the thyroid and decreases in TH levels (Zhang et al. 2018). Pinto et al. (2013) observed that exposure to TCS changed thyroid follicle morphology of adult zebrafish and increased the gene expression of the thyroid stimulating hormone (TSH) and the Na^+^-I^−^ symporter (NIS), which are involved in TH synthesis. Colloid accumulation in the thyroid tissue supports their hypothesis that TCS can affect TH synthesis and/or release, which is why there is an upregulation of TSH followed by hyperplasia in thyroid tissues. A decline in TH levels in zebrafish larvae after 14 d exposure to TCS was also detected by Tang et al. (2020), who also described hypotrophy and hyperplasia of thyroid follicles. Finally, the reduction of eye size in TCS-exposed zebrafish larvae confirmed the influence of THs on eye development in fish (Kim et al. 2018).

At present, OECD test guidelines for testing THSDCs in non-target organisms only cover tests with amphibians; comparable tests in fish have not yet been established. Research within the EU ERGO project has been directed to evaluate whether zebrafish can also be used as a model organism for THSDC testing (Holbech et al. 2020). Ideally, new endpoints can be implemented into OECD TG 236 (fish embryo toxicity test, FET), which is currently only used for the detection of acute toxicity in fish embryos. As an additional mechanistic tool, transgenic zebrafish lines expressing endocrine-regulated proteins tagged by fluorescent markers can be added to the test. This allows for the detection of different endocrine MoAs without using adult fish or expensive and time-consuming methods such as antibody staining (Rehberger et al. 2018). At OECD level, such tests with transgenic models have recently been established for the detection of estrogenic effects in fish as well as thyroid effects in amphibians (ECHA & EFSA 2018). In the present study, the transgenic zebrafish strain tg (*tg:mCherry*) developed by Opitz et al. (2012) was used to determine the effects of BP-2 and TCS on thyroid follicle development. This line expresses a membrane version of the red fluorescent protein mCherry specifically in thyrocyte membranes (Trubiroha et al. 2018), and allows improved tracking of thyroid follicle development in living zebrafish embryos.

The aim of this study was to demonstrate that eye histopathology, combined with the use of a transgenic thyroid zebrafish line within the setting of OECD TG 236 represents a simple tool for the assessment of THSDs in fish. Following 5 d exposure to TCS and BP-2, the thyroid follicles and the eyes were studied for morphological and histopathological changes in transgenic zebrafish embryos. Since eye development can also be affected by other MoAs such as neurotoxicity, changes in the thyroid follicles serve to document the specificity of effects in the eyes. As TCS and BP-2 have not been studied with respect to thyroid follicle and retinal layer morphology in zebrafish embryos, the study was also conducted to provide not only new insight into the MoAs but also to contribute to the environmental assessment of these substances.

## Materials and methods

### Test chemicals

Benzophenone-2 (BP-2, 2,2′,4,4-tetrahydroxybenzophenone, CAS 131–55-5) and triclosan (TCS, 2,4,4′-trichloro-2′-hydroxydiphenyl ether, CAS 3380–34-5) were purchased from Sigma Aldrich (Deisenhofen, Germany). BP-2 was dissolved in water, whereas TCS was dissolved in DMSO (dimethyl sulfoxide; CAS 67–68-5) with a final concentration of 0.02%. In addition, 300 µl 0.1 M NaOH per 50 ml test solution was used to increase the solubility of TCS. The sublethal treatment concentrations used in this study were based on previous range-finding FET experiments (LC_50_ for BP-2 and TCS: 26.3 mg/L and 217 µg/L, respectively). Since endocrine screening assays in fish should not be carried out above 1/10 of the LC_50_ (96 hpf) to avoid systemic toxicity effects (Wheeler et al. 2013), the following concentrations were tested: 0, 2, 4, and 7 mg/L for BP-2 and 0, 20, 40, and 80 µg/L for TCS (including a solvent control, which contained the same amount of DMSO and NaOH as the exposure groups). This approach represents the classic approach that is applied in regulatory testing of potential endocrine disruptors and does not necessarily reflect environmental relevance. All other chemicals were also purchased from Sigma unless stated otherwise.

### Zebrafish husbandry and exposure

A transgenic line of zebrafish expressing a fluorescent marker protein in the thyroid follicles tg(*tg:mCherry*; Opitz et al. 2012) kindly provided by the Costagliola laboratory at IRIBHM, in Brussels, Belgium was used to visualise thyroglobulin protein expression via fluorescence intensity. This transgenic line also lacks skin pigmentation to facilitate the detection of the thyroid follicles under the fluorescence microscope.

For egg production, adult zebrafish were kept in 15 L tanks in a water flow-through system (10% renewal per day) under continuous aeration with an artificial dark/light cycle of 10/14 h at 26 ± 1 °C. To guarantee for good water quality, conductivity (450–550 µS), pH (8.0–8.2), and oxygen saturation (90–95%) were monitored. Ammonia, nitrite, and nitrate were kept below recommended limits (0–5, 0.025–1, and 0–140 mg/L, respectively). In the morning, fish were fed *ad libidum* with fresh *Artemia* larvae (Great Salt Lake Artemia Cysts, Sanders, Ogden, USA); in the afternoon, they received dry flake food *ad libidum* (TetraMin™, Tetra-Werke, Melle). Excess food was removed by syphoning.

For the modified fish embryo toxicity test (FET; OECD 2013), artificial water (AW) was prepared according to OECD TG 203 (OECD 1992) and used for the preparation of the test solutions as well as a negative control (NC). The temperature was set at 26 ± 1 °C and the pH (7.75 ± 0.02) was adjusted with 0.1 M HCl and 0.1 M NaOH. The water was aerated for at least 20 min before use.

A standard FET was performed for concentration range-finding to ensure sublethal exposure concentrations in the subsequent modified FET for assessment of thyroid-related effects. Each compound was tested in a wide concentration range in 3 independent replicates to assess LC_50_ values. BP-2 was tested from 2 to 30 mg/L and TCS from 20 to 300 µg/L. Calculated LC_50_ values for BP-2 and TCS were 26.3 mg/L and 217 µg/L, respectively.

24 h before test initiation, the 24-well plates were pre-incubated with 2 ml/well of the TCS and BP-2 test solutions in an incubator at 26 ± 1 °C. For spawning, groups consisting of 8 male and 8 female zebrafish were transferred to custom-made, sloped spawning tanks with a gridded bottom. At the latest 1 h after fertilisation, eggs were collected, rinsed, and transferred to Petri dishes containing the test solutions. After checking for fertilisation or malformations, single eggs were transferred to fresh test solutions in the 24-well plates. Each treatment was performed in 3 independent replicates.

Embryos were raised in an incubator for 5 days with a 14/10 h day-night cycle at 26 ± 1 °C under semi-static exposure conditions: every morning an exposure solution renewal was performed, dead embryos were removed, and the remaining embryos were screened microscopically for malformations. A maximum mortality of 10% was considered as validity criterion for the test, as defined in OECD TG 236 (OECD 2013). Upon termination of exposure (5 dpf), embryos were prepared for further analysis under the fluorescent microscope.

### Analysis of thyroid follicles in transgenic zebrafish

For analysis of the thyroid follicles under the fluorescence microscope, embryos were mounted in a 3% methyl cellulose solution in AW (stirring for 48 h at 8 °C until transparency; storage at 8 °C). In order to allow for subsequent histological analysis of the eyes, embryos were sedated in 0.4% tricaine (MS-222) in a crystallisation dish immediately before use and only mounted for minimum periods of time: Using the tip of a micro loader, embryos were gently placed into a thin layer of methyl cellulose spread on a microscopy slide, and excess tricaine solution was absorbed with absorbent wipe (Kimtech) to prevent dilution of the methyl cellulose. Under a binocular, embryos were arranged in a line ventrally to the slide with the eyes oriented horizontally. To minimise the duration of anaesthesia, only 9 embryos were mounted on each slide. Based on prior statistical analyses, for the analysis of fluorescent thyroid follicles, at least 15 embryos per treatment were used. Not all embryos per treatment were analysed, as this would have significantly increased the time needed per exposure group, leading to developmental differences between groups that were analysed first and last, respectively. In case individuals did not show a fluorescence signal, these were excluded from analysis.

Micrographs of the embryos were taken at × 20 magnification on a Nikon Eclipse Ti-S inverted microscope connected to a Nikon DS-Fi3 camera (Nikon, Düsseldorf, Germany). Using the NIS-Elements 4.60 software, a Z-stack of the head region of each embryo was recorded, taking 9 subsequent images per channel every 2.5 μm. The TRITC filter was selected for *tg:mCherry* + */ − *with 500 ms exposure time, and brightfield images were taken using the 340 mm DIA channel with 20 ms exposure time. All images were analysed in greyscale using the open-source software Fiji ImageJ 2.1.0/1.53c/Java 1.8.0_172 (64-bit) to execute a self-written macro (details in supplements, Figure S1 and code S2). The parameter chosen for comparison of the thyroid follicles was the “integrated density” (ID), which is a product of the detected follicle area multiplied by the mean grey value of the selected grey area calculated by Fiji ImageJ.

### Preparation of the embryos for further histological analyses

After imaging of the thyroid follicles, embryos were carefully removed from the methyl cellulose and transferred to crystallisation dishes containing 0.4% tricaine, where remnants of methyl cellulose were rinsed off. For further histological analyses, all 24 embryos of one treatment were transferred into a 2 ml reaction tube. After removal of the tricaine solution, embryos were fixed for at least 12 h at 4 °C in 2 ml modified Davidson’s fixative.

Following fixation, embryos were rinsed three times in 70% ethanol and embedded in 4 groups of 6 embryos in 1.5% ultra PURE™ agarose (Life Technologies/ThermoFischer, Wiesbaden, Germany) in distilled water prepared according to the protocol of Sabaliauskas et al. (2006). The agarose matrixes were transferred into labelled Acetal Polymer embedding cassettes (Roth, Karlsruhe, Germany) and stored in 70% ethanol at 4 °C for at least 24 h and maximum 7 days. Using standard protocols, the agarose matrixes were dehydrated, infiltrated, and embedded in Paraplast at 60 °C (Leica, Nussloch, Germany) using a TP1020 tissue processor (Leica, Nussloch, Germany). The blocks with the agarose matrixes were stored at room temperature.

Coronal sections of the eyes of the embryos were taken with 4 µm thickness using a Reichert-Jung sledge microtome (Nussloch, Germany) with disposable MX35 Ultra low-profile microtome blades (34°/80 mm; Thermo-Fischer, Wiesbaden, Germany). The optical nerve served as a reference level for subsequent measurements of retinal layers. After mounting on a Superfrost® Plus microscopy slide (glass with positively charged surface; Menzel, Thermo-Fischer, Braunschweig, Germany), unstained sections were checked at 5 × magnification for orientation and quality under a light microscope (Axiostar plus, Zeiss, Oberkochen, Germany). After drying, standard haematoxylin and eosin staining using Mayer’s acid hemalum was performed (Mulisch & Welsch 2010).

The sections were analysed at × 40 magnification under a light microscope (Aristoplan Leitz, Wetzlar, Germany) equipped with a DFK 33UX264 digital camera. Micrographs were taken from coronal sections of at least one eye per embryo with the optical nerve longitudinally cut (in some cases, only one section was suitable for measuring). The thickness of the retina, the thickness of the retinal pigment epithelium (RPE), as well as the thickness of the photoreceptor layer (PRL) were measured (Figures S3 and S4) using the “Fiji” software by Schindelin et al. (2012). Since, in the case of the PRL, a difference in the ratio of the inner segment (IS) and the outer segment (OS) of the layer was observed, the OS of the PRL was also measured, and the ratio of the OS over the PRL was calculated as a percentage. For each parameter, the thickness was measured at eight different locations equally distributed over the section, and the mean was calculated. Areas artefactually modified were excluded from the analysis.

### Statistical analyses

Acute toxicities of TCS and BP-2 (LC_50_ at 120 hpf) were calculated using ToxRat 2.0 (ToxRat Solutions, Alsdorf, Germany).

For the analysis of the integrated density (follicle area multiplied by mean grey value) of the thyroid follicles as well as the stereological analysis of the retina, raw data were organised with Microsoft Excel 365 (Redmond, USA), and statistical analyses were performed using Prism 6 (Graph Pad, San Diego, USA). Statistical outliers were identified using robust regression and outlier removal (ROUT) with Q = 1%. Data were then tested for normal distribution using a Shapiro–Wilk normality test with α = 5%, followed by a one-way ANOVA with Dunnett’s multiple comparison test in case of normal distribution or a Kruskal–Wallis test followed by Tukey’s multiple comparison test in case of failure for normal distribution.

## Results

### Thyroid follicle size and staining intensity

**Fig. 1:**
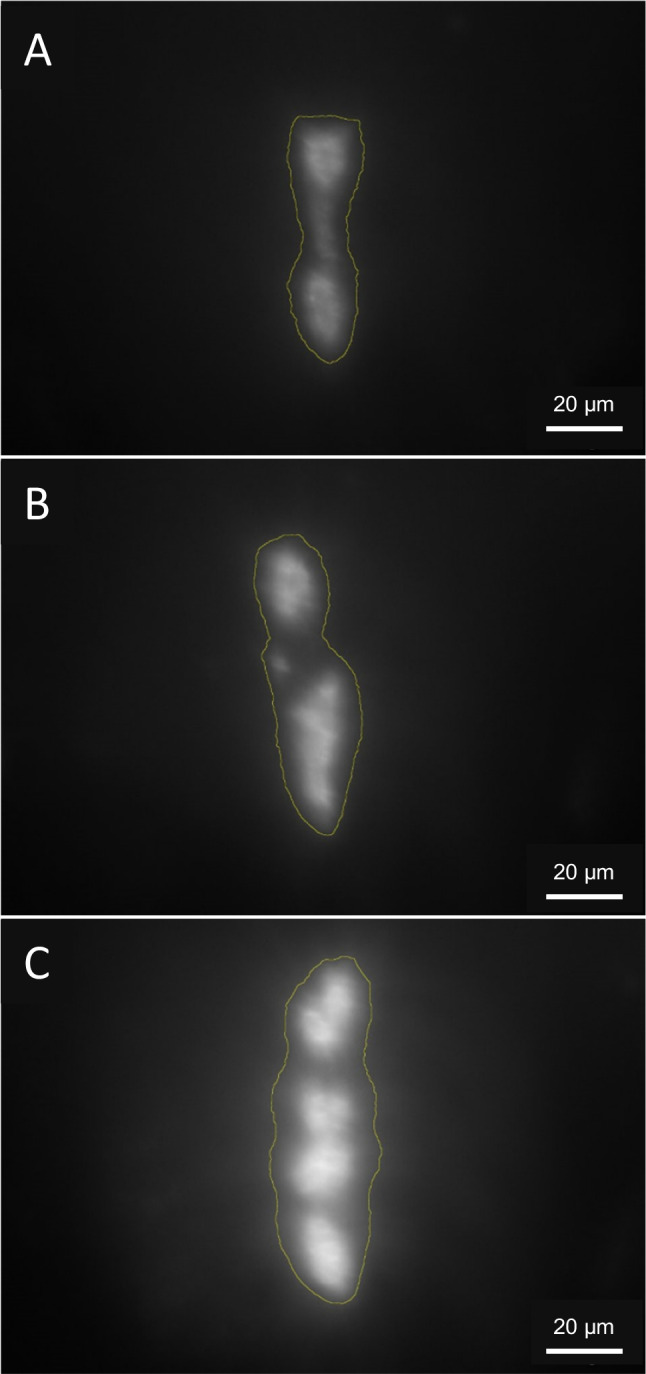
Head region of 5 d old zebrafish (*Danio rerio*) embryos from the negative control (**A**), embryos exposed to 4 mg/L benzophenone-2 (BP-2; **B**), and embryos exposed to 7 mg/L BP-2 (**C**). The micrographs show analysed areas of the greyscale version of the TRITC channel under the fluorescence microscope. An increase in size and intensity of the fluorescence signal after exposure to BP-2 compared to the solvent control (DMSO) could be observed.

**Fig. 2:**
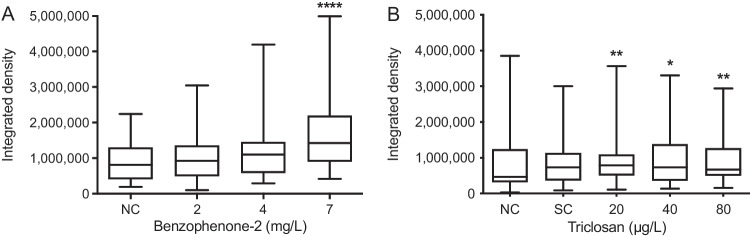
Integrated density of the thyroid follicles of zebrafish (*Danio rerio*) embryos after 5 days of exposure to benzophenone-2 (BP-2; **A**) and triclosan (TCS; **B**). BP-2: *n* = 45–47; TCS: *n* = 40–47. The central line displays the median and the whiskers are showing the standard deviation. Asterisks indicate statistically significant differences of the exposure groups to the negative control (NC) group: *p < 0.05, **p < 0.01; ****p < 0.0001. SC = solvent control

The size, number, and fluorescence intensity of follicles of BP-2-exposed zebrafish embryos increased with rising exposure concentrations (Fig. 1). This increase of integrated density became significant at 7 mg/L (Fig. 2A). Exposure to TCS caused significant differences between the negative control and all concentrations (Fig. 2B).

### Histopathology of the eyes in zebrafish embryos

**Fig. 3:**
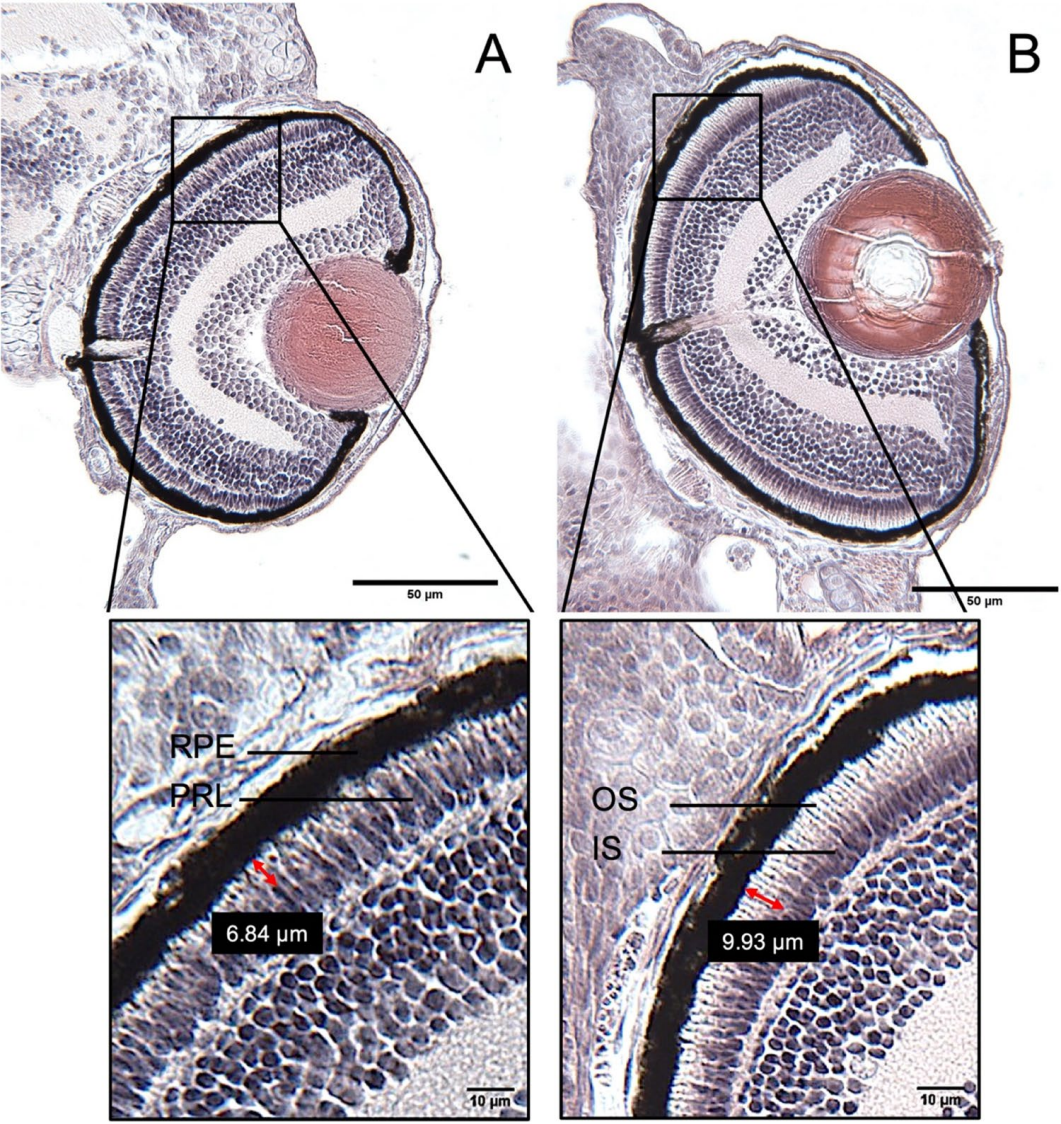
Changes of the thickness of the photoreceptor layer and its ratio according to the measurement of the outer segment (OS) of the photoreceptor layer (PRL) in the eyes of 5 d old zebrafish (*Danio rerio*) embryos after exposure to benzophenone-2 (BP-2). Paraffin-section with the visible optical nerve of 4 µm thickness and HE-stained. **A** Eye of an embryo of the negative control. **B** Eye of an embryo exposed to 7 mg/L of BP-2. An increase in the overall size of the PRL after treatment with BP-2 was obvious. Likewise, a change in the ratio of the outer and the inner segment (OS, IS) of the PRL is evident due to the increase in the thickness of the OS

**Fig. 4:**
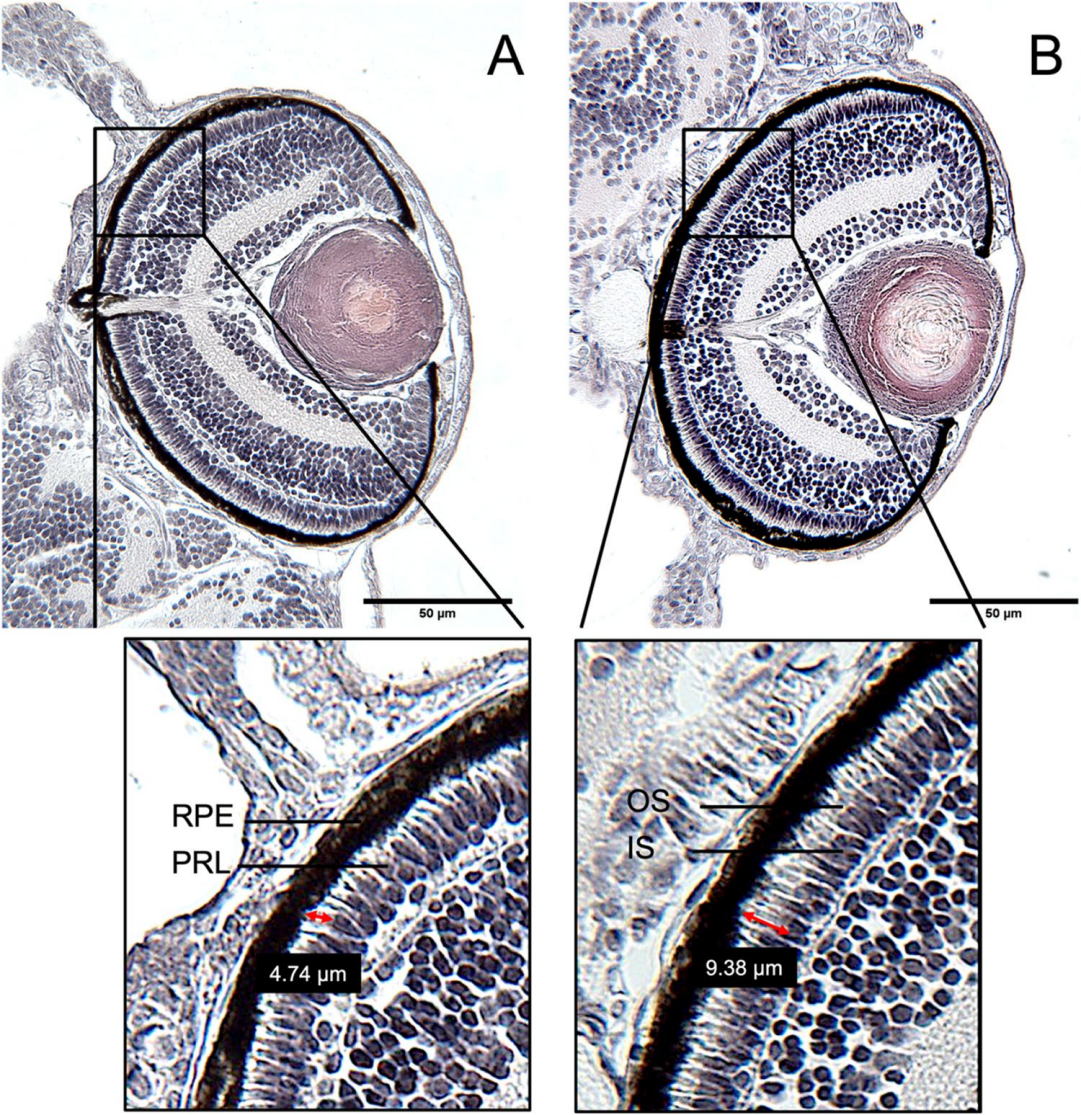
Changes of the outer and inner segment (OS/IS) ratio of the photoreceptor layer in the eyes of 5 days old zebrafish (*Danio rerio*) embryos exposed to triclosan (TCS). Paraffin-sections with the visible optical nerve of 4 µm thickness after HE-staining. **A** Eye section of an embryo of the negative control. **B** Eye section of an embryo exposed to 80 µg/L of TCS. An increase in the thickness of the OS of the PRL, associated with an increase of the OS/IS ratio of > 50% was evident

Example images of observed histology effects are presented in Fig. 3 (BP-2) and Fig. 4 (TCS).

**Fig. 5:**
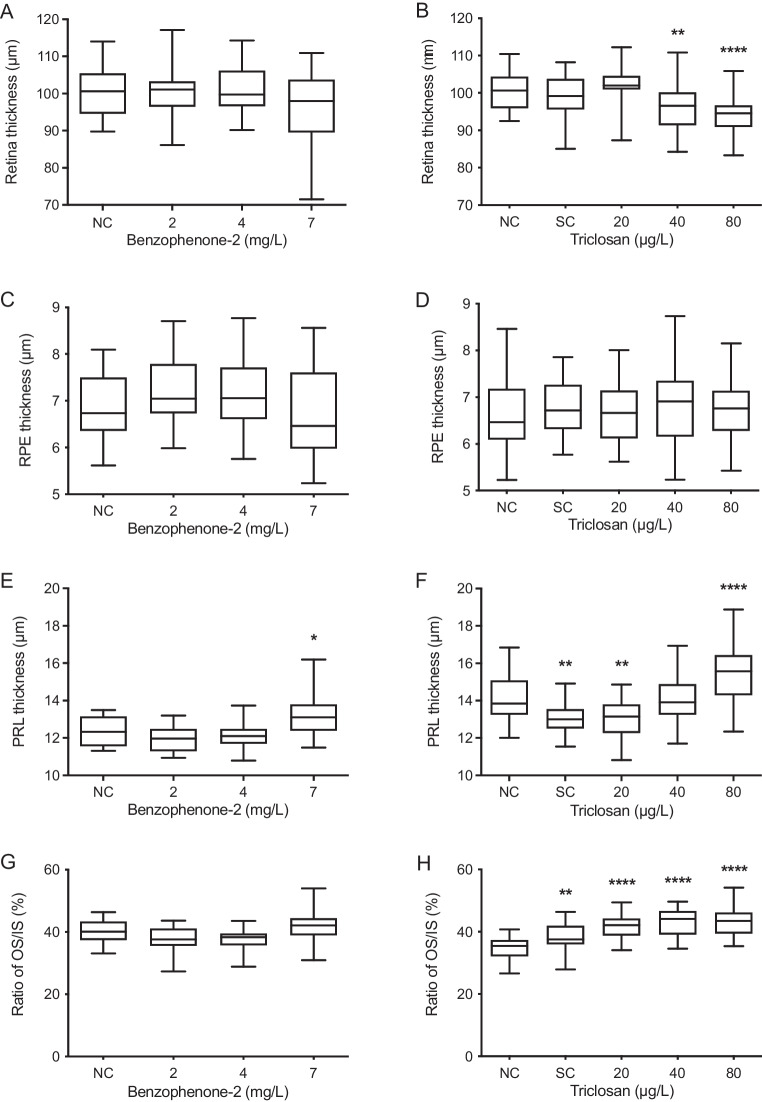
Quantitative changes in the morphology of various layers in the retina of eyes of 5 days old zebrafish (*Danio rerio*) embryos after exposure to benzophenone-2 (BP-2) or triclosan (TCS). **A**, **B** Thickness of the retina. **C**, **D** Thickness of the retinal pigment epithelium (RPE). **E**, **F** Thickness of the photoreceptor layer (PRL). **G**, **H** OS/IS ratios. BP-2: *n* = 21–33; TCS: *n* = 30–36. The central line displays the median and the whiskers are showing the standard deviation. Asterisks indicate statistically significant differences of the exposure groups to the negative control (NC) group. *p < 0.05, ****p < 0.0001. SC = solvent control

After BP-2 exposure, the thickness of the retina did not show any significant differences from the negative control (Fig. 5A); however, a minor decrease was visible after exposure to 7 mg/L BP-2. Likewise, the RPE did not show significant changes (Fig. 5C) but showed a slight increase with intermediate BP-2 concentrations (*p* = 0.2791 at 2 mg/L and 0.2577 at 4 mg/L). In contrast, the thickness of the PRL was significantly increased at 7 mg/L BP-2 (Fig. 5E), with values of 12.36 in controls and 13.21 µm in embryos exposed to 7 mg/L BP-2. The ratio between the OS and the IS did not show any significant change (Fig. 5G). The apparently minor increase at 7 mg/L BP-2 (*p* = 0.2151) was, however, clearly visible in the original micrographs of eye sections of BP-2-treated zebrafish embryos (Fig. 3).

The thickness of the retina of TCS-exposed embryos showed a significant decrease in treatments with 40 µg/L and 80 µg/L TCS (Fig. 5B; S5). Whereas the RPE did not reveal any significant differences (Fig. 5D), the PRL thickness showed a significant decrease in the DMSO (solvent control/SC) group and the embryos exposed to 20 µg/L TCS (Fig. 5F). At 80 µg/L TCS, however, a significant increase of the PRL thickness was observed. The OS/IS ratio in the PRL was consistently increased over negative controls (Figs. 4 and 5H), with the strongest differences for the 80 µg/L embryos (43.14 µm versus 34.58 µm in negative controls). Since both thickness and OS/IS ratio of the PRL were also altered in the SC, additional statistical analyses were made in which exposure groups were compared to the SC to check for DMSO-induced effects (data not shown). The analyses revealed that the highest exposure group (80 µg/L TCS) had a significantly increased PRL thickness compared to the SC. Also, the OS/IS ratio was significantly higher in all TCS exposure groups than in the SC.

## Discussion

As a part of the EU H2020-project ERGO, which aims to identify new thyroid-specific endpoints for implementation into current test guidelines for the detection of endocrine disruption in fish (Holbech et al. 2020), the present study was designed to elucidate the adverse effects by two environmental THSDCs, benzophenone-2 (BP-2) and triclosan (TCS) on the development of thyroid follicles and eyes of zebrafish embryos. Results demonstrate that both compounds had an impact on thyroid follicle morphology and eye morphology of exposed embryos. These findings provide further evidence for the impact of THSDCs on the eye development of fish, which could serve a relevant endpoint in future testing of chemicals.

### Effects of benzophenone-2 and triclosan on thyroid follicle development

Image analysis of the thyroid follicles of the transgenic line revealed a compensatory reaction of the thyroid tissue in 5 d old zebrafish embryos after exposure to either TCS or BP-2, as indicated by elevated integrated density (ID) values under the fluorescence microscope. The ID integrates the size of the thyroid follicles and fluorescence intensity and covers both cellular proliferation (hyperplasia) and enlargement of the thyrocytes (hypertrophy). Higher ID values do not only reflect morphological alterations but mirror changes in the functional status of follicles following disruption by THSDCs. Since fluorescence intensity reflects thyroglobulin expression, a stronger signal from the follicles suggests an induction of synthesis and enhanced secretion of thyroglobulin into the colloid. Hypertrophy of thyroid follicles, e.g., an increase in the height of the follicular cells, has been shown to be directly proportional to the release of TSH (Carr and Patiño, 2011). Therefore, the follicle ID can be regarded as indicator for thyrocyte activity and in turn provide evidence for the potential effects on TH levels.

The hyperplastic effect of BP-2 was directly evident from fluorescence micrographs and showed a conspicuous positive dose–response relationship (cf. Figures 1 and 2A). Compensatory hyperplasia of the thyroid follicles as observed in the present study is consistent with previous studies showing that BP-2 exposure leads to decreased T3 and T4 levels (Schmutzler et al. 2007). The decline in TH levels is most likely a consequence of an inhibition of thyroid peroxidase (TPO), which is responsible for the iodination of thyroglobulin (Song et al. 2012). TPO inhibition by BP-2 likely results in a decrease of TH synthesis and a reduced activation of thyroid receptors (THRs), which then triggers a feedback reaction: the release of TSH by the pituitary stimulates increased thyroid follicle activity and TH synthesis, which is reflected by the increase in the number and/or size of thyroid follicle cells. However, other modes-of-action of BP-2 cannot be excluded, such as inhibition of deiodinase enzymes as it was reported for the TPO inhibitor propylthiouracil (PTU, Stinckens et al. 2020).

Likewise, TCS induced a significant increase in the size and fluorescence intensity of the thyroid follicles in all exposure concentrations (cf. Figure 2B), yet without a clear dose–response relationship. This in line with previous studies by Pinto et al. (2013), who described thyroid hyperplasia in combination with elevated transcript levels of NIS and TSH after TCS exposure of adult zebrafish. NIS inhibition would lead to decreasing T3 and T4 levels, as it is the case with PTU (De Sandro et al. 1991), a typical TPO inhibitor. Again, to compensate low levels of T3 and T4, more TSH would be released by the pituitary, resulting in compensatory induction of NIS expression. Elevated NIS activities would stimulate iodine uptake by thyrocytes and increased loading of thyroglobulin with oxidised iodine by TPO, which might eventually compensate levels of T3 and T4. An increased turnover of thyroglobulin could well explain the increasing fluorescence intensity in the thyroid follicles in the present study. Since, however, the exact MoAs of BP-2 and TCS are still not fully understood, further analyses at the molecular level are required.

### Effects of benzophenone-2 and triclosan on eye development

Since a functional visual system is of utmost importance for fish survival, alterations of eye development at both molecular and morphological levels might be useful as endpoints close to apical, population-relevant adverse effects of THSDC exposure (Baumann et al. 2016 & 2019; Dang et al. 2021; Gölz et al. 2022). As the most promising parameters, alterations on the thickness of the entire retina and the thickness of the retinal pigment layer (RPE) and the photoreceptor Layer (PRL) were measured.

Albeit statistically not significant, a subtle non-monotonic response of the RPE with a transient increase of the thickness followed by a decrease at the highest BP-2 concentration was observed. A reduction in RPE thickness in THSDC-exposed zebrafish embryos was also detected in other studies performed in our laboratory with TBBPA and PTU (Baumann et al. 2016). For BP-2, no further information is available for effects on the RPE, but other studies into THSDCs with a similar MoA as BP-2 (TPO inhibition) showed similar effects in zebrafish (Baumann et al. 2016; Avallone et al. 2015). The sequence of events leading from TPO inhibition to altered retinal structures has formed the basis for an AOP on THSDC-triggered eye disruption in (zebra) fish (Gölz et al. 2022). For BP-2, only the thickness of the PRL showed a statistically significant change at the highest concentration of 7 mg/L. In fact, BP-2 is known to reduce T3 and T4 levels in zebrafish embryos via TPO inhibition (Song et al. 2012; Schmutzler et al. 2007). Since THRs are located in the outer nuclear layer of the retina in fish (Betrand et al. 2007) and THs regulate cone development in the PRL by interacting with the THRs (Suzuki et al. 2013), a decrease in T3 and T4 levels due to exposure to BP-2 in developing zebrafish embryos could lead to altered PRL cell height.

If compared to BP-2, TCS-exposed zebrafish embryos showed much stronger histological alterations in the eyes, as was already evident from the significant decrease in retina thickness. This is in line with observations by Kim et al. (2018), who described a smaller eye diameter in zebrafish exposed to TCS. A decrease in eye diameter was also evident in zebrafish embryos exposed to other TPO inhibitors such as PTU (Baumann et al. 2016, Reider and Connaughton 2014) and could be correlated to impaired foraging efficiency of zebrafish embryos after exposure to TCS (Wirt et al. 2018). Whereas the thickness of the RPE did not respond to TCS exposure, changes in PRL thickness showed a monotonic increase over water and DMSO controls with increasing TCS concentrations. However, it needs to be noted that also DMSO had a significant effect on PRL thickness. The underlying mechanism of this effect is not clear. Toxic effects of DMSO can likely be excluded, as the chosen concentration of 0.02% is in the range that is accepted within OECD TG 236 (FET) and embryos did not show any signs of toxicity. The effects of DMSO on eye development have not been reported in the literature.

Since TCS exposure did not result in a reduction of the RPE thickness, an indirect damage to the PRL via the RPE can likely be excluded. Rather, the increase in the OS/IS ratio indicates an increase of the OS of the PRL relative to a decrease of the IS, which represent the active part of the PRL, i.e., the area, in which the cell bodies with organelles are located. The functional implications of this shift between portions of the photoreceptors are not clear, but previous studies show that the phototransduction process might be directly impaired by THSDC exposure (Baumann et al. 2019; Bagci et al. 2015). Again, the observed effect of DMSO on this parameter remains unclear but needs to be taken into account in future testing when this solvent is needed.

The underlying reason for this abnormal development could be that trß2, a TH-dependent receptor that is responsible for the differentiation of the cones in the PRL (Deveau et al. 2020), could be influenced by the reduced T3 and T4 levels after BP-2 exposure. Gan and Novales Flamarique (2010) showed that THs accelerate opsin expression in differentiating cones and induce the opsin switch, a shift from the expression of UV opsin to blue opsin. In their study, the authors observed that THSDCs, for example, PTU could repress this opsin switch. DuVal and Allison (2018) investigated the role of trß in cone differentiation and found that its knockdown caused almost complete absence of red cones and an increase in UV cone abundance.

Morphological changes of the PRL were also observed in studies with other THSDCs. For instance, Xu et al. (2015) detected a failure of forming the OS in the PRL after exposure to 2,2′,4,4′-tetrabromdiphenyl ether (BDE47), an observation in line with results from studies in rat eyes (Gamborino et al. 2001). Other studies revealed that TCS is responsible for an increase in TSH and NIS gene transcripts (Pinto et al. 2013). With the hypothesis that TSH production is indirectly stimulated by TCS due to the inhibition of the NIS symporter, TCS could lead to a downregulation of gene expression of THRs like PTU (Baumann et al. 2016), which might explain the changes in the differentiation of the PRL.

The effects on eye development observed after exposure to BP-2 and TCS might have serious consequences for developing fish since changes in the affected cell layers might directly affect vision as a physiological capacity vital for the survival of the fish. In fact, changes in the retinal structures of zebrafish embryos have been shown to lead to modified responses to light (Houbrechts et al. 2016), and our own behavioural studies detected differences in swimming activities of zebrafish embryos after exposure to PTU (Baumann et al. 2016). In a previous study, exposure to tetrabromobisphenol A and PTU caused impaired vision, which led to decreased activity of zebrafish embryos (Baumann et al. 2016). Since the correct visual function is an indispensable prerequisite for successful foraging and predator avoidance (Fuiman et al. 2006), such changes in fish behaviour are likely to directly lead to reduced survival in the wild (Besson et al. 2020; Dehnert et al. 2019) and, thus, to adverse effects at the population level (Rearick et al. 2018).

### Applicability of the transgenic zebrafish test system

The present report also represents a case study for demonstration of the applicability of the transgenic zebrafish test system for detection of thyroid-related effects in fish. Results indicate that the use of the transgenic line for thyroid follicle analyses in combination with histopathological analysis of eye development is a promising approach that covers both mechanistic aspects and endpoints close to apical parameters such as survival or population performance, both of which are relevant for regulatory purposes. For demonstration of THSDC impact on the thyroid as the major target organ, the transgenic fish line allows standard image analysis of fluorescent follicles as a relatively simple, cheap, and reproducible method. In existing amphibian and mammalian test systems, detection of morphological changes in the thyroid gland represents a well-established parameter for the detection of endocrine activity (EFSA 2018). In fact, numerous previous studies showed that thyroid follicle histopathology is a very sensitive and suitable method for the detection of thyroid-related effects in fish. Many studies identified hyperplasia and hypertrophy of the thyroid follicles in zebrafish after exposure to other THSDCs using classic histopathology methods (Schmidt and Braunbeck 2011 & 20,12; van der Ven et al. 2006; Pinto et al. 2013; Patiño et al. 2003).

Classic histopathological methods, however, are more time- and labour-intensive than the use of transgenic reporter systems. The transgenic line used in the present study has already been used for the demonstration of effects on follicle development (Opitz et al. 2012) such as hyperplastic effects from exposure to THSDCs like resorcinol and potassium perchlorate (Jarque et al. 2018). Similarly, Trubiroha et al. (2018) observed an increased number of thyroid cells in zebrafish exposed to PTU. Not only transgenic zebrafish lines can be used for detection of effects on the THS, also a transgenic line of *Xenopus laevis* is used for THSDC assessments with amphibians (Couderq et al. 2020). The size of the epithelium cells, which is also included in the analysis with the transgenic fish, is considered to best represent the functional status of the follicles (Carr & Patiño, 2011). Consequently, an increased size of the thyroid follicular cells indicates a higher activity in producing THs and vice versa (Eales & Brown, 1993). Direct measurements of TH levels would be needed to fully confirm this correlation, but the biological evidence can be considered strong.

The use of embryonic life stages has many practical advantages such as the short exposure duration and the limited space needed. However, also ethical considerations based on the 3R principles can be met by using embryos instead of adult fish (Strähle et al. 2012). The main motivation to use older life stages and longer exposure tests is to cover apical, population-relevant endpoints like sexual development in order to assess the ecotoxicological impact of a chemical. Such apical endocrine-related endpoints are not established in tests with fish embryos, which is why our approach using eye development might serve as a starting point for the establishment of a rapid, simple, and cost-effective, but meaningful test system. As outlined above, functional optical sense can be considered highly population-relevant, as impaired visual function will most certainly decrease survival performance of populations.

## Conclusions

The purpose of the present study was to investigate the effects of benzophenone-2 (BP-2) and triclosan (TCS) on the morphology of thyroid follicles and the development of the eyes in transgenic zebrafish embryos after 5 days of exposure. Results clearly document that both compounds affect the HPT axis of zebrafish by causing morphological alterations in the thyroid follicles and in the retinal layers of the eye. The simultaneous occurrence of effects in both organ systems makes a functional relationship likely. Therefore, eye development may be considered as a suitable endpoint for TH system disruption in fish. Although the present study documented the direct impact of BP-2 and TCS on the TH system, for a more-in-depth understanding of their MoAs and a comprehensive verification of the ecological relevance of the alterations observed, further studies are required in areas ranging from experiments at the molecular level to behavioural assessment. Moreover, mixture effects of potential THSDCs should be implemented in analyses to increase the environmental relevance.

In conclusion, our case study demonstrates that morphological changes of thyroid follicles and retinal layers can be detected for different THSDCs in a comparably short and easy test system. In a simplified way, our test system represents an AOP-like approach that covers mechanistic endpoints and endpoints close to apical parameters for the assessment of potential THSDCs. The present study shows that the used methods as well as the used test organism are suitable for establishment in test guidelines.

## Supplementary Information

Below is the link to the electronic supplementary material.

Supplementary file1 (DOCX 5757 KB)

## Data Availability

The datasets used and/or analysed during the current study are available from the corresponding author on reasonable request.
