# Supplementary material for: Developmental exposure to triclosan and benzophenone-2 causes morphological alterations in zebrafish (Danio rerio) thyroid follicles and eyes
Source: Environ Sci Pollut Res Int. 2022 Dec 10;30(12):33711–24. doi: 10.1007/s11356-022-24531-2 (PMC9736712; doi:10.1007/s11356-022-24531-2)
Supplement: Supplementary file 1 — Supplementary file1 (DOCX 5757 KB) [file 11356_2022_24531_MOESM1_ESM.docx]

**Supplementary Information:**

**Title:** Developmental exposure to triclosan and benzophenone-2 causes morphological alterations in zebrafish (*Danio rerio*) thyroid follicles and eyes

**Authors:** Kraft, Maximilian; Goelz, Lisa; Rinderknecht, Maximilian; Koegst, Johannes; Braunbeck, Thomas; Baumann, Lisa *

* Corresponding author: lisa.baumann@uni-heidelberg.de

Aquatic Toxicology and Ecology Section, Centre of Organismal Studies, University of Heidelberg, Im Neuenheimer Feld 504, 69120 Heidelberg, Germany

**Figure S1:** Visualization of the Fiji ImageJ analyses with an embryo from the negative control (20× magnification). A: Brightfield (DIA 340 mm) image of the head region. B: The TRITC channel as grayscale as it was used for the image analysis. C: Picture after the analysis. Three pictures with average intensity were overlaid and the area within the yellow circles was analyzed.


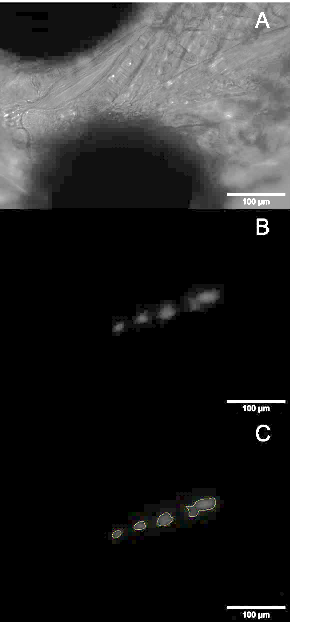


**S2: Code of the ImageJ macro**

input = getDirectory("");

output = "C:\Users\johan\Desktop\Uni Kl\ImageJ_Analysed\09.05.21\NK";

list = getFileList(input);

setBatchMode(true);

for (i = 0; i < list.length; i++) {

run("Bio-Formats Importer", "open=["+input+list[i]+"] autoscale color_mode=Default rois_import=[ROI manager] view=Hyperstack stack_order=XYCZT");

name = getTitle();

Stack.setChannel(1);

run("Reduce Dimensionality...", "slices keep");

name2 = getTitle();

//selectWindow("06.07.PTU150_-1.nd2");

run("Z Project...", "start=4 stop=6 projection=[Average Intensity]");

//run("Subtract Background...", "rolling=50");

run("Gaussian Blur...", "sigma=3.5");

setAutoThreshold("Otsu dark");

//run("Threshold...");

//setThreshold(26, 255);

setOption("BlackBackground", true);

run("Convert to Mask");

run("Analyze Particles...", "display exclude clear summarize add in_situ");

saveAs("Results", ""+output+name+"_Summary.csv");

//Table.rename("Summary", "Results")

selectWindow("Results");

saveAs("Results", ""+output+name+"_Results.csv");

selectWindow(name2);

run("Z Project...", "projection=[Average Intensity]");

roiManager("Show All without labels");

run("Flatten");

saveAs("Tiff", ""+output+name+"_overlay.tif");

roiManager("reset");

run("Clear Results");

close();

close();

close();


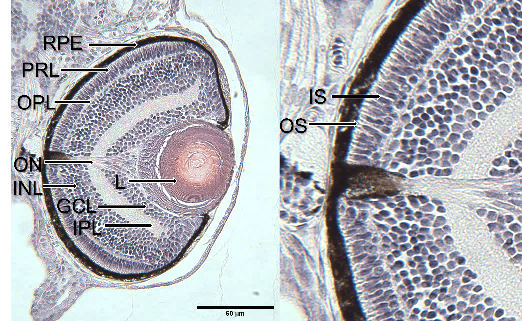


**Figure S3:** Histological section of the eye of a 5 d old zebrafish (*Danio rerio*) embryo. Hematoxylin and eosin (HE) staining. Abbreviations: RPE − retinal pigment epithelium, PRL − photoreceptor layer, OS − outer segment of the PRL, IS − inner segment of the PRL, ON − optical nerve, L − lens.


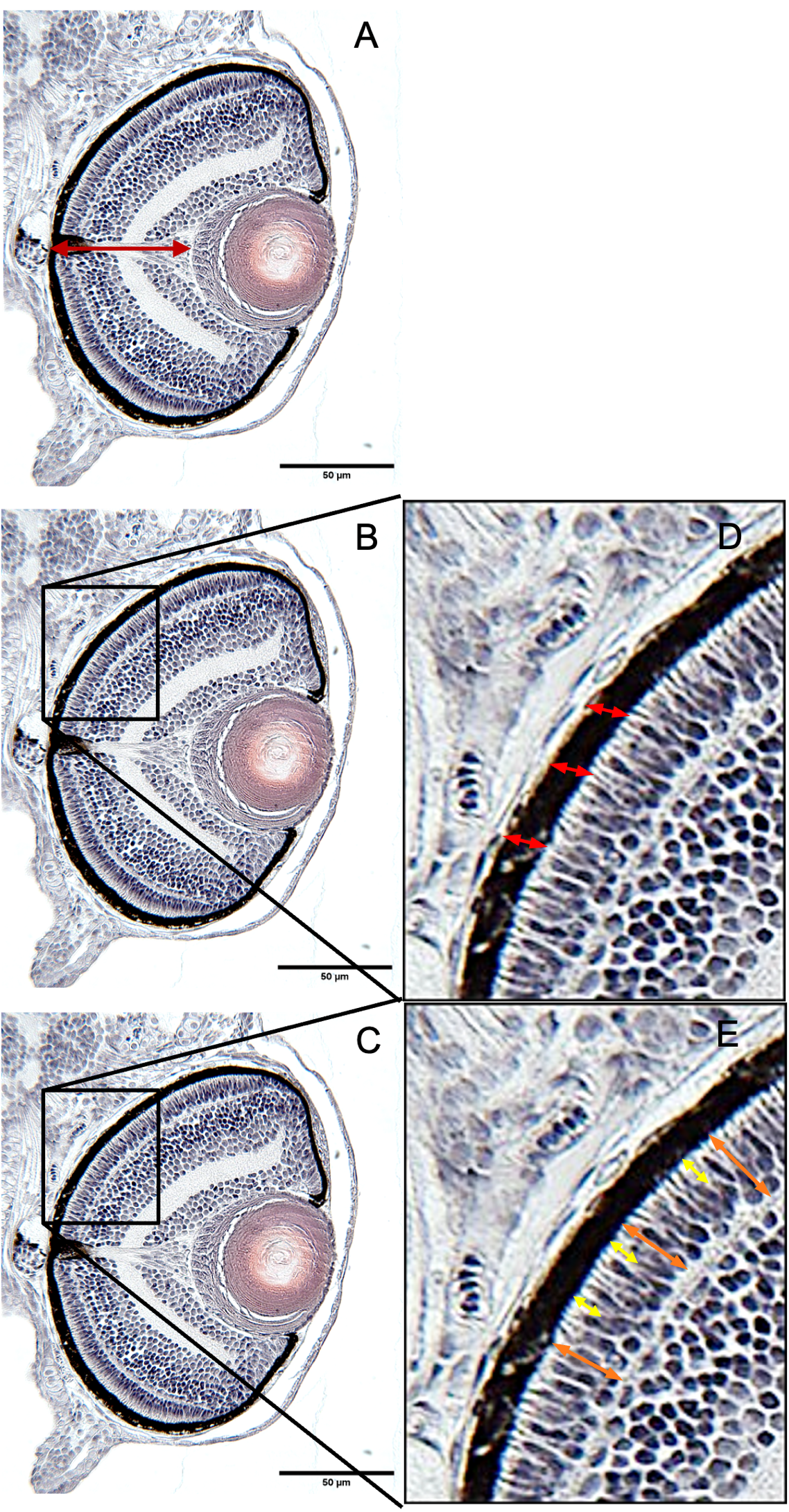


**Figure S4:** Overview of the different measurements in eye sections of 5 d old zebrafish (*Danio rerio*) embryos. A: Measurement of the thickness of the retina; B: measurement of the thickness of the retinal pigment epithelium; C: measurement of the thickness of the photoreceptor layer and its outer segment.


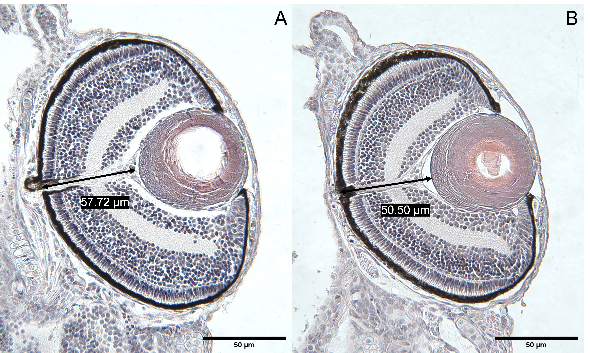


**Figure S5:** Changes in the thickness of the retina of 5 d old zebrafish (Danio rerio) embryos exposed to triclosan (TCS). Paraffin-section with the visible optical nerve of 4 µm thickness and HE-stained. A: eye of an embryo of the negative control; B: eye of an embryo exposed to 80 µg/L of TCS. A decrease of the whole eye size was observed.
